# Level and correlates of physical activity and sedentary behavior in patients with type 2 diabetes: A cross-sectional analysis of the Italian Diabetes and Exercise Study_2

**DOI:** 10.1371/journal.pone.0173337

**Published:** 2017-03-14

**Authors:** Stefano Balducci, Valeria D’Errico, Jonida Haxhi, Massimo Sacchetti, Giorgio Orlando, Patrizia Cardelli, Nicolina Di Biase, Lucilla Bollanti, Francesco Conti, Silvano Zanuso, Antonio Nicolucci, Giuseppe Pugliese, Italian Diabetes and Exercise Study 2 (IDES_2) Investigators

**Affiliations:** 1 Department of Clinical and Molecular Medicine, ‘‘La Sapienza” University, Rome, Italy; 2 Diabetes Unit, Sant’Andrea Hospital, Rome, Italy; 3 Metabolic Fitness Association, Monterotondo, Rome, Italy; 4 Department of Human Movement and Sport Sciences, ‘‘Foro Italico” University, Rome, Italy; 5 Laboratory of Clinical Chemistry, Sant’Andrea Hospital, Rome, Italy; 6 Diabetes Unit, Fatebenefratelli San Pietro Hospital, Rome, Italy; 7 Centre for Applied Biological & Exercise Sciences, Faculty of Health & Life Sciences, Coventry University, Coventry, United Kingdom; 8 Center for Outcomes Research and Clinical Epidemiology (CORE), Pescara, Italy; Vanderbilt University, UNITED STATES

## Abstract

**Objective:**

Patients with type 2 diabetes usually show reduced physical activity (PA) and increased sedentary (SED)-time, though to a varying extent, especially for low-intensity PA (LPA), a major determinant of daily energy expenditure that is not accurately captured by questionnaires. This study assessed the level and correlates of PA and SED-time in patients from the Italian Diabetes and Exercise Study_2 (IDES_2).

**Methods:**

Three-hundred physically inactive and sedentary patients with type 2 diabetes were enrolled in the IDES_2 to be randomized to an intervention group, receiving theoretical and practical exercise counseling, and a control group, receiving standard care. At baseline, LPA, moderate-to-vigorous-intensity PA (MVPA), and SED-time were measured by accelerometer. Physical fitness and cardiovascular risk factors and scores were also assessed.

**Results:**

LPA was 3.93±1.35 hours∙day^-1^, MVPA was 12.4±4.6 min∙day^-1^, and SED-time was 11.6±1.2 hours∙day^-1^, with a large range of values (0.89–7.11 hours∙day^-1^, 0.6–21.0 min∙day^-1^, and 9.14–15.28 hours∙day^-1^, respectively). At bivariate analysis, LPA and MVPA correlated with better cardiovascular risk profile and fitness parameters, whereas the opposite was observed for SED-time. Likewise, values of LPA, MVPA, and SED-time falling in the best tertile were associated with optimal or acceptable levels of cardiovascular risk factors and scores. At multivariate analysis, age, female gender, HbA_1c_, BMI or waist circumference, and high-sensitivity C reactive protein (for LPA and SED-time only) were negatively associated with LPA and MPA and positively associated with SED-time in an independent manner.

**Conclusions:**

Physically inactive and sedentary patients with type 2 diabetes from the IDES_2 show a low level of PA, though values of LPA, MVPA, and SED-time vary largely. Furthermore, there is a strong correlation of these measures with glycemic control, adiposity and inflammation, thus suggesting that even small improvements in LPA, MVPA, and SED-time might be associated with significant improvement in cardiovascular risk profile.

**Trial registration:**

ClinicalTrials.gov NCT01600937

## Introduction

Physical inactivity, i.e. an insufficient amount of physical activity (PA) according to current guidelines, and sedentary behavior, i.e. a large amount of time spent in a sitting or reclining posture, have become the major determinants of the epidemic of obesity and type 2 diabetes. Recent data from the National Health and Nutrition Examination Survey (NHANES) indicate that the dramatic rise in the prevalence of obesity and abdominal adiposity among US adults from 1988 to 2010 was associated with an increased proportion of subjects reporting no leisure-time PA, but not with changes in average caloric intake [1]. Another report from the NHANES (1988–2008) shows that less than 50% of US adults achieve the recommended PA level and ~30% of them engage in no PA at all, though projections to 2020 suggest some improvement [2]. The prevalence of physical inactivity is even higher in individuals with type 2 diabetes or in those at highest risk for developing this condition. In fact, in the Medical Expenditure Panel Survey, only 39% of adults with diabetes were physically active versus 58% of those without diabetes and the proportion of active adults without diabetes declined as the number of risk factors increased [3]. Physical inactivity is also a major predictor of all-cause mortality in subjects with type 2 diabetes [4–6].

However, recent findings from individuals with type 2 diabetes indicate a significant association between sedentary (SED)-time and metabolic risk, independently of several confounders, including time spent in moderate-to-vigorous-intensity PA (MVPA). Indeed, MVPA was not associated with clustered metabolic risk after accounting for SED-time [7, 8]. These reports are consistent with previous studies showing a positive relationship between daily sitting time or television viewing with all-cause and cardiovascular mortality [9, 10] as well as cardiovascular risk factors [11, 12]. Taken together, the above observations support the concept that physically inactivity and sedentary behavior influence health outcomes through distinct pathways and highlight the need to target both conditions. In fact, the recommended amount of daily MVPA (30 min) [13] represents less than 5% of the time spent awake. Thus, additional efforts should be made to reduce sedentary behaviors during the rest of the day by promoting light-intensity PA (LPA), which is a major determinant of total daily energy expenditure and may be increased more easily than MVPA in patients with type 2 diabetes [14, 15]. A recent report from the Australian Diabetes, Obesity, and Lifestyle (AusDiab) study showed that sitting-reduction strategies targeting increased standing, stepping, or both, may benefit cardio-metabolic health [16].

Although PA/exercise is now considered as a cornerstone of prevention and management of type 2 diabetes [17, 18], it is difficult to put into action PA/exercise recommendations for a number of barriers [19]. In particular, compliance is usually poor, since sustained changes in PA may require much time and effort. In fact, both randomized controlled trials (RCTs) and observational studies have shown that health benefits from PA/exercise may require large volumes [20–22]. Nevertheless, in the long-term, even smaller volumes of daily PA may provide beneficial effects on glycemic control [23] and mortality [5, 24, 25]. Increasing total daily unstructured PA, mainly LPA, as suggested by current guidelines [13], may also reduce metabolic and cardiovascular risk in patients with type 2 diabetes by decreasing the amount of SED-time [14,15].

While the time spent in MVPA is usually low in subjects with type 2 diabetes, the level of LPA and, conversely, the amount of SED-time may vary greatly, even in patients classified as physically inactive and sedentary according to current definitions. However, both LPA and SED-time are not accurately captured by questionnaires [26] and, hence, reliable data in patients with type 2 diabetes are lacking. In subjects without diagnosed diabetes from the AusDiab study, LPA was 5.8 hours∙day^-1^, MVPA was 36.0 min∙day^-1^, and SED-time was 8.4 hours∙day^-1^, on average, as measured objectively by accelerometer [27].

This study aimed at assessing the level and correlates of LPA, MVPA, and SED-time in physically inactive and sedentary patients with type 2 diabetes participating in the Italian Diabetes and Exercise Study_2 (IDES_2).

## Subjects and methods

In this cross-sectional analysis, we used the data collected at the baseline visit for the IDES_2. This is an open-label, parallel RCT aimed at assessing the efficacy of a behavioral intervention strategy, as compared with standard medical care, in increasing total daily PA and reducing SED-time (primary endpoint) in patients with type 2 diabetes, as previously detailed [28].

### Ethics

The study complies with the Declaration of Helsinki.

The research protocol, which follows the SPIRIT guideline, was approved by the Ethics Committee of Sant'Andrea Hospital (Prot. n. 212/2012) and written informed consent was provided by each participant.

### Participants

The main entry criterion was known type 2 diabetes of at least 1-year duration. Additional requirements were age 40–80 years, BMI 27–40 kg/m^2^, physical inactivity (i.e. less than 150 min∙week^-1^ of moderate to vigorous aerobic exercise spread out over at least 3 days during the week, with no more than 2 consecutive days between bouts of aerobic activity) [29], and sedentary lifestyle (i.e. more than 8 hours∙day^-1^ spent in any waking behavior characterized by an energy expenditure ≤1.5 metabolic equivalents [METs]) [30] from at least 6 months, ability to walk 1.6 Km without assistance, and eligibility after cardiologic evaluation.

The study was conducted in three tertiary referral outpatients Diabetes Clinics in Rome. From October 2012 to February 2014, three-hundred patients were recruited to be randomized 1:1 to an intervention (INT) group (n = 150), receiving theoretical [21] and practical [31] exercise counseling on top of standard care, and a control (CON) group (n = 150), receiving only standard care including general physician recommendations for daily PA [28].

### Measurements

At baseline, all patients were asked to wear an accelerometer for objectively assessing PA and SED-time. They also underwent assessment of physical fitness and measurement of anthropometrical, clinical and biochemical parameters. Data on socio-demographic characteristics of study subjects and presence of complications were also collected.

#### Assessment of PA and SED-time

At screening, PA level was evaluated retrospectively using the Minnesota LTPA questionnaire [28]. Each participant was then outfitted with a uni-axial piezoelectric accelerometer MyWellness Key (Technogym, Cesena, IT) [32], which offers the possibilities of storing 30 days of continuous movement detection and provides measures of the minutes spent at light, moderate and vigorous intensities and the total volume of PA, which are aligned with other laboratory validations [33, 34]. This device was recently shown to measure PA volume accurately and to acceptably discriminate between LPA and moderate intensity PA in individuals with type 2 diabetes [35]. Each participant wore the device for seven consecutive days. Upon waking, immediately after bathing or showering, participants were asked to attach the device at the waistband in midline of the right anterior hip and to wear it all day (except if swimming) up to bedtime. Patients were also asked to report on a daily diary the hours spent wearing the instrument, sleeping and snapping, and performing non-accelerometer recordable PAs such as swimming, cycling, skiing, etc.

The time the patient was awake and was not wearing the accelerometer was assumed to be spent in sedentary activities (e.g. taking a shower, getting dressed), unless the participant reported in the diary PAs which cannot be performed while wearing the accelerometer (e.g. swimming). SED-time was then calculated by adding this time to that recorded by the accelerometer with readings <100 counts∙min^-1^, a threshold which corresponds with sitting, reclining, or lying down, i.e. to <1.5 METS [28].

Matthews’ cut-points were used to identify time spent in LPA (100–1951 counts∙min^-1^ corresponding to 1.5–2.9 METs), whereas Freedson’s cut-points were used to determine time spent in moderate intensity (1952–5724 counts∙min^-1^ corresponding to 3–5.9 METs) and vigorous intensity (>5725 counts∙min^-1^ corresponding to >6 METs) PA. Time spent in non-accelerometer recordable PAs, as reported on the daily diary, was added to that recorded by the accelerometer. Moderate intensity PA was combined with vigorous intensity PA into MVPAs, as participants spent little time in vigorous intensity PA [28].

#### Assessment of physical fitness

Physical fitness was assessed by evaluating cardio-respiratory fitness, strength, and flexibility. The tests were preceded by two consecutive run-in sessions to become familiar with testing devices and protocols.

Cardio-respiratory fitness was assessed by a maximal treadmill exercise test using a Balke protocol and expressed as maximal oxygen uptake (VO_2max_), as previously detailed [28]. Isometric muscle strength was measured by means of a strain gauge tensiometer (Digimax, Mechatronic GmbH, Germany) [36]. Lower limb muscle strength was assessed by maximal voluntary contractions (MVCs) performed at a costumed leg extension machine (Leg press, Easy Line, Technogym), with a 90° angle at the knee and the hip. Upper body muscle strength was assessed by MVCs performed at a shoulder press (Shoulder press/Lat pull, Easy Line, Technogym) along the sagittal plane, with a 90° and 45° angle at the elbow and between the upper arm and the trunk, respectively. For each exercise, three MVC were performed, with 3 min rest interval between contractions. For hip and trunk flexibility assessment, a standard bending test in the standing position was executed [28]. The test was performed three times and the distance between the finger and the ground was measured at the third attempt.

#### Assessment of cardiovascular risk factors and scores

All patients underwent a structured interview in order to collect the following information: age, smoking status, known diabetes onset and duration, history of cardiovascular disease and microvascular complications, current treatments including glucose-, BP- and lipid-lowering drugs.

Body weight and height were measured using scale and stadiometer and BMI was then calculated as weight (kg)∙height^-2^ (m^-2^), while waist circumference was taken at the umbilicus. Body composition was evaluated by assessing fat mass and fat-free mass by the use of a bio-impedance device (Tanita BF664, Vernon Hills, IL, USA). Then, BP was recorded with a sphygmomanometer after a five-minute rest with the patient seated with the arm at the heart level.

Biochemical tests were centralized at the Laboratory of Clinical Chemistry of Sant’Andrea Hospital, an accredited and ISO9001 certified structure. The following parameters were assessed using standard analytical techniques [28]: HbA_1c_, fasting plasma glucose (FPG), serum insulin, triglycerides, total, LDL and HDL cholesterol, high sensitivity-C-reactive protein (hs-CRP), serum creatinine, and albumin:creatinine ratio (ACR) on first-voided urine samples. Then, the Homeostasis Model Assessment-Insulin Resistance (HOMA-IR) index was calculated from FPG and insulin levels; estimated glomerular filtration rate (eGFR) was computed from serum creatinine by the use of the Chronic Kidney Disease Epidemiology Collaboration equation; and global and fatal coronary heart disease (CHD) and stroke 10-year risk scores were calculated using the United Kingdom Prospective Diabetes Study (UKPDS) risk engine, as previously detailed [28].

### Statistical analysis

For the purpose of this analysis considering only baseline data, patients subsequently assigned to the INT and CON groups were pooled together. Data are expressed as mean ± SD or number of cases (percentage). The χ^2^ test for categorical variables and the Student’s t test or the corresponding nonparametric Mann-Whitney test for continuous variables were utilized to compare clinical characteristics between males and females. One-way ANOVA or the corresponding nonparametric Kruskal-Wallis test were used to evaluate changes of measured variables with tertiles of LPA, MPVA, and SED-time.

Bivariate analyses of correlations between LPA; MVPA, or SED-time and physical fitness parameters and cardiovascular risk factors and scores were performed using Spearman’s rho. Partial correlation was also applied for adjusting each bivariate correlation for confounders. Then, multivariate regression analyses with stepwise backward selection of variables were applied to assess the independent correlates of LPA, MVPA, or SED-time. Covariates were age, gender, diabetes duration, HbA_1c_, HOMA-IR, BMI, fat mass, waist circumference, systolic BP, hs-CRP, eGFR, and ACR. Additional regression models were applied by including socio-demographic features or presence of long-term complications.

Statistical analyses were performed at the CORE using SAS software release 9.3 (Cary, NC, USA).

## Results

### Clinical characteristics of study subjects

Patients from the IDES_2 cohort had a mean age of 61.6 years (SD 9.9), a mean diabetes duration of 10.5 years (SD 8.0), and a male-to-female ratio of 61/39. The clinical features of study subjects are reported in Table 1.

**Table 1:** Clinical features, physical fitness parameters, and PA and SED-time values in the whole cohort and by gender.

Variable	Total	Males	Females	*P*
**n (%)**	300 (100)	184 (61.3)	116 (38.7)	
**Age, years**	61.6±9.9	61.0±9.8	62.6±10.0	0.163
**Smoking, n (%)**				<0.0001
**Never**	122 (40.7)	41 (22.3)	81 (69.8)	
**Former**	121 (40.3)	101 (54.9)	20 (17.2)	
**Current**	57 (19.0)	42 (22.8)	15 (12.9)	
**Diabetes duration, years**	10.5±8.0	10.5±8.3	10.6±7.7	0.711
**HbA**_**1c**_**, % (nmol·mol**^**-1**^**)**	7.38±1.49	7.33±1.41	7.45±1.61	0.518
	(57.1±16.3)	(56.6±15.4)	(57.9±17.6)	
**FPG, mmol·l**^**-1**^	7.56±2.73	7.47±2.48	7.70±3.09	0.969
**Insulin, pmol∙l**^**-1**^	89.3±86.2	86.7±91.3	93.4±77.6	0.280
**HOMA-IR**	4.48±5.60	4.44±6.41	4.54±4.00	0.292
**BMI, kg·m**^**-2**^	30.0±5.1	29.1±4.5	31.4±5.6	<0.0001
**Fat mass, %**	31.7±10.2	25.8±7.6	40.9±6.0	<0.0001
**Fat-free mass, kg**	56.5±11.3	63.1±8.2	45.9±6.6	<0.0001
**Waist circumference, cm**	103.6±12.8	103.1±12.5	104.5±13.2	0.375
**Triglycerides, mmol·l**^**-1**^	1.82±1.39	1.79±1.61	1.87±0.94	0.049
**Total cholesterol, mmol·l**^**-1**^	4.67±1.01	4.55±0.98	4.86±1.02	0.009
**HDL cholesterol, mmol·l**^**-1**^	1.22±0.36	1.15±0.34	1.34±0.36	<0.0001
**LDL cholesterol, mmol·l**^**-1**^	2.89±0.87	2.90±0.92	2.89±0.77	0.931
**Systolic BP, mmHg**	140.1±20.4	138.9±18.6	142.0±23.0	0.193
**Diastolic BP, mmHg**	82.9±11.7	83.0±11.3	82.7±12.3	0.859
**hs-CRP, mg·l**^**-1**^	4.99±8.82	3.59±4.37	7.21±12.79	0.004
**eGFR, ml·min**^**-1**^**·1.73 m**^**-2**^	87.1±18.4	87.7±18.1	86.2±19.0	0.509
**ACR, mg·g**^**-1**^	73.4±332.7	84.3±401.0	56.2±177.4	0.132
**UKPDS CHD 10-year risk score**	20.6±13.8	24.7±14.8	14.3±9.0	<0.0001
**UKPDS fatal CHD 10-year risk score**	15.1±12.8	17.8±14.1	10.7±8.8	<0.0001
**UKPDS stroke 10-year risk score**	13.4±12.7	14.7±14.0	11.2±9.8	0.086
**UKPDS fatal stroke 10-year risk score**	2.18±2.44	2.33±2.58	1.94±2.18	0.211
**VO**_**2max**_**, ml∙min**^**-1**^**∙kg**^**-1**^	24.7±6.5	26.8±5.9	21.2±5.7	<0.0001
**Upper body strength, Nm**	254.8±92.5	299.4±80.3	184.1±61.6	<0.0001
**Lower body strength, Nm**	161.1±60.4	188.8±53.9	117.3±41.1	<0.0001
**Bending, cm**	16.7±11.7	18.1±11.0	14.5±12.4	0.008
**LPA, hours∙day**^**-1**^	3.93±1.35	4.20±1.36	3.50±1.24	<0.0001
**MVPA, min∙day**^**-1**^	12.4±4.6	13.8±4.2	10.1±4.4	<0.0001
**SED-time, hours∙day**^**-1**^	11.6±1.2	11.4±1.2	11.9±1.2	<0.0001

Values are mean±SD for continuous variables and n (%) for categorical variables.

PA = physical activity; SED-time = sedentary time; FPG = fasting plasma glucose; HOMA-IR = Homeostasis Model Assessment-Insulin Resistance; BP = blood pressure; hs-CRP = high sensitivity-C-reactive protein; eGFR = estimated glomerular filtration rate; ACR = albumin:creatinine ratio; UKPDS = United Kingdom Prospective Diabetes Study; CHD = coronary heart disease; VO_2max_ = maximal oxygen uptake; LPA = light intensity PA; MVPA = moderate-to-vigorous intensity PA.

On average, they showed a reasonably good, though not optimal, control of glucose levels (mean HbA_1c_ 7.38%) as well as of the other cardiovascular risk factors, with the possible exception of adiposity level and distribution, and a significant 10-year risk of CHD (>20%).

As previously reported [37], the cardiovascular risk profile was worse in females than in males, though the UKPDS CHD risk scores remained higher in men. Medication use is reported in S1 Table and was similar between males and females for most agents.

### Level of PA and SED-time

As expected, physical fitness parameters indicated a low fitness and accelerometer measures showed a low amount of both LPA (mean value 3.93 hours∙day^-1^) and MVPA (mean value 12.4 min∙day^-1^) and a high level of SED-time (mean value 11.6 hours∙day^-1^) (Table 1).

**Fig 1:**
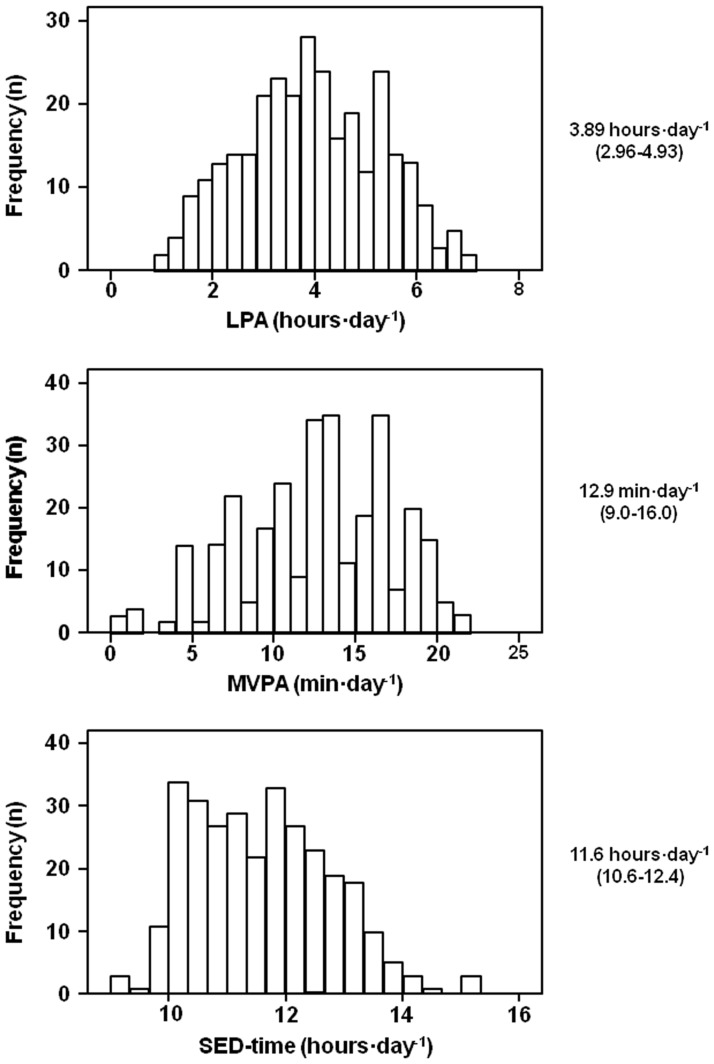
Distribution of LVPA, MVPA, and SED-time values in the study subjects. Median (interquartile range) for each variable are reported on the right. LPA = light intensity physical activity; MVPA = moderate-to-vigorous intensity; SED-time = sedentary time.

However, even if per protocol all patients were physically inactive and sedentary, the range of LPA (0.89–7.11 hours∙day^-1^), MVPA (0.6–21.0 min∙day^-1^), and SED-time (9.14–15.28 hours∙day^-1^) values were quite wide (Fig 1).

### Correlates of PA and SED-time

Bivariate correlations (Table 2) showed negative associations of both LPA and MVPA with age, diabetes duration, HbA_1c_, FPG, HOMA-IR, BMI, fat mass, waist circumference, hs-CRP, UKPDS risk scores, VO_2max_, strength, and SED-time. Only LPA correlated with ACR and bending, whereas only MVPA correlated with fat-free mass, triglycerides, systolic blood pressure, and eGFR. In contrast, SED-time was positively associated with age, diabetes duration, HbA_1c_, FPG, insulin, HOMA-IR, BMI, fat mass, waist circumference, triglycerides, systolic BP, hs-CRP, ACR, UKPDS risk scores, and physical fitness measures. When partial correlation analysis was applied to adjust for confounders, only age, HbA_1c_, BMI or fat mass, and hs-CRP remained significantly associated with LPA, MVPA and SED-time.

**Table 2:** Bivariate correlations of LVPA, MVPA, and SED-time with cardiovascular risk factors and scores and physical fitness parameters (Spearman’s rho).

Variable	LPA		MVPA		SED-time	
	R	*P*	R	*P*	R	*P*
**Age**	-0.198	0.001	-0.281	<0.0001	0.152	0.008
**Diabetes duration**	-0.153	0.008	-0.178	0.002	0.167	0.004
**HbA**_**1c**_	-0.305	<0.0001	-0.196	0.001	0.323	<0.0001
**FPG**	-0.222	<0.0001	-0.144	0.012	0.218	<0.0001
**Insulin**	-0.109	0.059	-0.100	0.084	0.139	0.016
**HOMA-IR**	-0.180	0.002	-0.137	0.018	0.207	<0.0001
**BMI**	-0.248	<0.0001	-0.218	<0.0001	0.225	<0.0001
**Fat mass**	0.243	<0.0001	-0.351	<0.0001	0.227	<0.0001
**Fat-free mass**	0.077	0.183	0.205	<0.0001	-0.071	0.224
**Waist circumference**	-0.230	<0.0001	-0.214	<0.0001	0.200	<0.0001
**Triglycerides**	-0.111	0.055	-0.127	0.027	0.135	0.019
**Total cholesterol**	0.031	0.596	0.074	0.199	-0.026	0.657
**HDL cholesterol**	0.048	0.403	0.021	0.717	-0.066	0.253
**LDL cholesterol**	0.069	0.233	0.136	0.018	-0.074	0.202
**Systolic BP**	-0.089	0.124	-0.177	0.002	0.116	0.045
**Diastolic BP**	-0.003	0.956	0.001	0.975	0.028	0.628
**hs-CRP**	-0.226	<0.0001	-0.256	<0.0001	0.202	0.001
**eGFR**	0.093	0.110	0.136	0.018	-0.041	0.479
**ACR**	-0.155	0.007	-0.035	0.547	0.136	0.018
**UKPDS CHD risk score**	-0.162	0.005	-0.118	0.042	0.142	0.014
**UKPDS fatal CHD risk score**	-0.206	<0.0001	-0.189	0.001	0.187	0.001
**UKPDS stroke risk score**	-0.182	0.002	-0.239	<0.0001	0.148	0.010
**UKPDS fatal stroke risk score**	-0187	0.001	-0.256	<0.0001	0.161	0.005
**VO**_**2max**_	0.586	<0.0001	0.663	<0.0001	-0.522	<0.0001
**Upper body strength**	0.281	<0.0001	0.397	<0.0001	-0.235	<0.0001
**Lower body strength**	0.341	<0.0001	0.412	<0.0001	-0.299	<0.0001
**Bending**	-0.143	0.013	-0.107	0.064	0.142	0.014
**LPA**	-	-	0.591	<0.0001	-0.855	<0.0001
**MVPA**	0.591	<0.0001	-	-	-0.547	<0.0001
**SED-time**	-0.855	<0.0001	-0.547	<0.0001	-	-

LPA = light intensity physical activity; MVPA = moderate-to-vigorous intensity; SED-time = sedentary time; FPG = fasting plasma glucose; HOMA-IR = Homeostasis Model Assessment-Insulin Resistance; BP = blood pressure; hs-CRP = high sensitivity-C-reactive protein; eGFR = estimated glomerular filtration rate; ACR = albumin:creatinine ratio; UKPDS = United Kingdom Prospective Diabetes Study; CHD = coronary heart disease; VO_2max_ = maximal oxygen uptake.

Levels of HbA_1c_, FPG, HOMA-IR, BMI, fat mass, waist circumference, hs-CRP, ACR, UKPDS risk scores, and physical fitness parameters significantly improved with tertiles of LPA and worsened with tertiles of SED-time (except for ACR). A trend similar to that of LPA was observed with tertiles of MVPA, except for a significant decrease of systolic blood pressure, but not of FPG, ACR, total CHD risk score, and bending, and an increase in fat-free mass (Table 3).

**Table 3:** Cardiovascular risk factors and scores and physical fitness parameters by tertiles of LVPA, MVPA, and SED-time.

Variable	Tertiles of LPA (hours∙day^-1^)				Tertiles of MVPA (min∙day^-1^)				Tertiles of SED-time (hours∙day^-1^)			
	I (<3.25)	II (3.25–4.57)	III (>4.57)	*P*	I (<10.8)	II (10.8–15.0)	III (>15.0)	*P*	I (<10.9)	II (10.9–12.1)	III (>12.1)	*P*
	2.42±0.61	3.90±0.35	5.46±0.61		7.0±2.5	12.5±1.0	17.3±1.7		10.3±0.4	11.5±0.4	12.9±0.7	
**HbA**_**1c**_**, %**	7.93±1.62	7.38±1.40	6.82±1.21	<0.0001	7.63±1.61	7.50±1.45	7.02±1.34	0.009	6.79±1.22	7.33±1.29	8.00±1.67	<0.0001
**(nmol·mol**^**-1**^**)**	(63.2±17.7)	(57.2±15.3)	(51.0±13.2)		(59.9±17.6)	(58.5±15.8)	(53.2±14.6)		(50.7±13.3)	(56.6±14.1)	(63.9±18.3)	
**FPG, mmol·l**^**-1**^	8.19±2.92	7.69±2.82	6.80±2.24	<0.0001	8.01±3.10	7.80±2.80	6.91±2.13	0.243	6.88±2.24	7.44±2.50	8.35±3.19	0.002
**Insulin, pmol·l**^**-1**^	97.5±97.9	99.9±92.6	70.6±61.5	0.012	95.3±81.6	91.6±88.0	81.6±89.0	0.059	73.1±63.8	96.7±94.3	98.2±95.5	0.060
**HOMA-IR**	5.35±7.03	5.05±5.79	3.05±2.97	<0.0001	4.93±4.96	4.78±6.41	3.77±5.33	0.426	3.20±3.10	4.75±5.78	5.49±7.02	0.003
**BMI, kg·m**^**-2**^	31.5±5.5	29.8±4.9	28.7±4.4	<0.0001	31.6±5.5	29.6±5.1	28.9±4.3	<0.0001	28.3±4.1	30.8±5.3	31.0±5.4	<0.0001
**Fat mass, %**	34.2±10.5	32.0±10.0	28.8±9.4	0.001	36.3±10.3	31.1±9.6	27.8±8.8	<0.0001	27.5±8.7	34.4±10.2	33.1±10.3	<0.0001
**Fat-free mass, kg**	55.8±11.5	56.1±11.8	57.5±10.7	0.518	54.0±11.7	56.1±12.0	59.1±9.8	0.006	58.0±10.7	55.1±11.0	56.2±12.2	0.194
**Waist circumference, cm**	107.3±14.0	102.7±12.0	100.9±11.4	0.001	107.6±14.6	102.1±11.5	101.4±11.3	0.001	99.9±10.8	105.3±11.9	105.7±14.7	0.002
**Systolic BP, mmHg**	143.5±22.4	137.4±16.6	139.4±21.5	0.095	144.1±21.8	140.1±20.2	136.3±18.7	0.025	137.7±19.3	137.7±17.6	144.8±23.3	0.016
**Diastolic BP, mmHg**	83.6±14.0	82.1±10.0	82.9±10.7	0.678	83.5±13.8	82.5±11.7	82.6±9.4	0.797	82.3±9.8	82.4±10.1	84.0±14.5	0.512
**hs-CRP, mg·l**^**-1**^	6.52±9.32	5.72±11.39	2.72±3.15	0.001	6.62±10.96	5.06±6.83	3.38±7.96	<0.0001	2.82±3.51	5.22±9.61	6.92±11.02	0.001
**eGFR, ml·min**^**-1**^**·1.73 m**^**-2**^	84.2±21.1	88.5±16.4	88.7±17.2	0.153	84.2±19.9	87.0±18.3	89.9±16.8	0.089	88.5±17.6	87.8±17.3	85.0±20.2	0.364
**ACR, mg·g**^**-1**^	116.1±521.6	65.1±158.6	39.1±184.7	0.009	109.2±524.2	54.5±151.1	57.6±198.2	0.841	48.5±192.0	50.7±136.3	121.1±524.9	0.086
**UKPDS CHD 10-year risk score**	24.4±14.4	19.1±14.0	18.4±12.3	0.002	21.4±12.5	21.9±16.5	18.7±11.9	0.381	19.3±12.8	18.1±12.7	24.5±15.1	0.005
**UKPDS fatal CHD 10-year risk score**	18.9±13.4	13.7±12.8	12.5±11.3	<0.0001	16.4±11.5	16.3±15.3	12.6±11.0	0.037	13.2±11.8	13.1±11.6	18.9±14.1	0.001
**UKPDS stroke 10-year risk score**	16.5±13.8	12.8±13.1	10.7±10.2	0.002	15.7±12.8	13.8±14.1	10.7±10.6	0.003	11.6±11.1	12.4±12.2	16.1±14.2	0.030
**UKPDS fatal stroke 10-year risk score**	2.80±2.83	1.97±2.09	1.77±2.23	0.001	2.67±2.60	2.20±2.42	1.70±2.21	0.001	1.84±2.29	1.91±1.91	2.78±2.91	0.013
**VO**_**2max**_**, ml∙min**^**-1**^**∙kg**^**-1**^	20.4±6.0	24.5±5.1	29.1±5.0	<0.0001	19.8±5.4	24.5±4.6	29.3±5.5	<0.0001	28.7±5.1	24.2±5.5	21.0±6.2	<0.0001
**Upper body strength, Nm**	225.1±86.9	254.9±90.6	284.4±91.3	<0.0001	215.6±86.1	257.0±93.2	289.7±83.7	<0.0001	284.3±85.6	246.7±90.6	233.5±94.5	<0.0001
**Lower body strength, Nm**	136.5±49.2	160.7±61.4	186.2±59.7	<0.0001	132.9±51.3	163.4±56.2	185.5±61.3	<0.0001	184.0±59.4	161.8±61.4	137.6±51.2	<0.0001
**Bending, cm**	19.7±12.0	14.5±11.6	15.9±11.0	0.004	17.4±12.7	17.1±12.1	15.7±10.4	0.559	15.8±11.0	14.2±11.4	20.2±11.9	0.001

Values are mean±SD. LPA = light intensity physical activity; MVPA = moderate-to-vigorous intensity; SED-time = sedentary time; FPG = fasting plasma glucose; HOMA-IR = Homeostasis Model Assessment-Insulin Resistance; BP = blood pressure; hs-CRP = high sensitivity-C-reactive protein; eGFR = estimated glomerular filtration rate; ACR = albumin:creatinine ratio; UKPDS = United Kingdom Prospective Diabetes Study; CHD = coronary heart disease; VO_2max_ = maximal oxygen uptake.

At multivariate analysis, age, female gender, HbA_1c_, BMI (for LPA) or waist circumference (for MVPA and SED-time), and hs-CRP (for LPA and SED-time only) were negatively associated with LPA and MPA and positively associated with SED-time in an independent manner (Table 4). The total fraction of variance in LPA, MPA, and SED-time accounted for by variables included in multiple regression models were 22.7%, 28.4%, and 21.0%, respectively. Results did not change when socio-demographic characteristics or presence of complications were included in the models. These variables were not or minimally associated with LPA, MPA, and SED-time when analyzed in regression models including only age and gender (not shown).

**Table 4:** Independent correlates of PA and SED-time (multiple regression analysis with stepwise backward selection of variables).

Variable	LPA		MVPA		SED-time	
	Beta	*P*	Beta	*P*	Beta	*P*
**Age**	-0.175	0.001	-0.262	<0.0001	0.144	0.006
**Female gender**	-0.323	0.001	-0.332	<0.0001	0.137	0.011
**HbA**_**1c**_	-0.246	<0.0001	-0.165	0.001	0.310	<0.0001
**BMI**	-0.326	0.001	-	-	-	-
**Waist circumference**	-	-	-0.155	0.002	0.122	0.022
**hs-CRP**	-0.135	0.013	-	-	0.177	0.001

PA = physical activity; SED-time = sedentary time; LPA = light intensity PA; MVPA = moderate-to-vigorous intensity PA; hs-CRP = high sensitivity-C-reactive protein.

## Discussion

This work provides, for the first time, an objective picture of the level of PA and sedentary behavior in patients with type 2 diabetes willing to participate in a research-based PA trial and identifies the main correlates of LPA, MVPA, and SED-time in these individuals.

### Level of PA and SED-time

The main findings of this study are the low level of PA / high level of sedentary behavior in patients with type 2 diabetes and the large variability of accelerometer measures in individuals who are classified as physically inactive and sedentary according to current definitions. In fact, on average, study subjects showed accelerometer-derived measures of LPA, MVPA, and SED-time which were worse than those observed in nondiabetic individuals from the AusDiab [27]. Moreover, half of participants in the IDES_2 fell in a range LPA (2.96–4.93 hours∙day^-1^), MVPA (9.0–16.0 min∙day^-1^), and SED-time (10.6–12.4 hours∙day^-1^) far from the thresholds indicated by current guidelines [13]. These observations indicate that there is a large room for action in order to increase PA level and to decrease SED-time to the recommended levels in patients with type 2 diabetes.

The very strong correlation between LPA and SED-time confirms the close inverse association between the two parameters and supports the recommendation to increase not only MVPA, but also LPA, in order to contrast the independent deleterious effect of sedentary behavior on health outcomes [13]. As suggested by others [14, 15], increasing LPA might represent the first step toward the adoption of a physically active lifestyle, as it would reduce SED-time and increase physical function, thus allowing patients to subsequently engage in MVPA.

Some subjects were found to engage in virtually no PA and to spend almost all the time in a sitting or reclining position, whereas some other individuals approached the recommended levels. This finding suggests that prescription of PA/exercise to patients with type 2 diabetes should be preceded by an accurate evaluation of behavior of each individual, with particular reference to the amount of daily unstructured PAs and time spent in sitting or reclining position, in order to provide patients with adequate and specific recommendations.

### Correlates of PA and SED-time

The close associations of accelerometer measures with virtually all metabolic parameters and cardiovascular risk factors and scores support the importance of increasing LPA (and MVPA) and concurrently decreasing SED-time. In particular, this analysis highlights the link between PA behavior and glycemic control, adiposity and inflammation, with HbA_1c_, BMI or waist circumference, and hs-CRP correlating independently with LPA, MVPA, and SED-time, together with age and gender. These observations are consistent with the recognized role of physically inactivity and sedentary behavior in the ongoing epidemic of obesity and type 2 diabetes [1,2]. They are also in agreement with current guidelines considering PA/exercise as a cornerstone in the prevention and management of type 2 diabetes [13] as well as with the anti-inflammatory effect of PA/exercise [38], which was shown to reduce markers of inflammation and insulin resistance, independent of weight loss [39].

More importantly, these data indicate that small improvements in LPA, MVPA, and SED-time might be associated with better glycemic control and cardiovascular risk profile, even if absolute values remain quite distant from recommended levels. In fact, values of LPA, MVPA, and SED-time falling in the best tertile (i.e. >4.57 hours∙day^-1^, >15.0 min∙day^-1^, and <10.86 hours∙day^-1^, respectively) were shown to be associated with optimal or acceptable levels of metabolic parameters and cardiovascular risk factors and scores, whereas the opposite was observed for values in the worst tertiles. These findings support the concept that even limited behavioral changes with small volumes of daily PA, mainly of low intensity, may provide beneficial effects in patients with type 2 diabetes [5, 23–25], thus suggesting the need to encourage all patients to be more active, while setting more or less ambitious goals based on the individual attitude and compliance. However, due to the cross-sectional nature of this analysis, this hypothesis needs to be confirmed by intervention studies, such as the IDES_2. In fact, a poor cardiovascular risk profile might be the cause, rather than the effect, of physical inactivity and/or sedentary behavior, though the beneficial impact of increasing PA and reducing sedentary SED-time on glycemic control and modifiable cardiovascular risk factors is well-established.

### Strengths and limitations

A major strength of our study is that this is the first report on objective measures of PA and SED-time in a large contemporary cohort of patients with type 2 diabetes, very well characterized for cardio-metabolic risk profile and physical fitness level.

One limitation is the cross-sectional design, which does not allow assessing cause-effect relationships. Finally, the IDES_2 cohort is not fully representative of patients with type 2 diabetes, as subjects having any condition limiting/contraindicating PA or achieving the recommended level of PA were excluded. However, this analysis was specifically aimed at assessing the PA and SED-time profile of physically inactive and sedentary patients with type 2 diabetes, who represent the vast majority of the individuals suffering from this condition and those who are candidate to receive (and may benefit from) a behavioral intervention for adopting a physically active lifestyle.

### Conclusions

In conclusion, the objective assessment of PA behavior by accelerometer in physically inactive and sedentary patients with type 2 diabetes from the IDES_2 showed that level of PA is low in these individuals, though values of LPA, MVPA, and SED-time vary largely.

Furthermore, there is a strong correlation between these measures and cardio-metabolic risk profile, with an independent association with HbA_1c_, BMI or waist circumference, and hs-CRP, in addition to age and gender.

## Supporting information

S1 File IDES_2 Investigators. Diabetes Clinics; Metabolic Fitness Centers; Central laboratory; Data management team; Steering Committee.
(DOCX)

S1 Table Medication use in the whole cohort and by gender. (DOCX)

## References

[1] Ladabaum U, Mannalithara A, Myer PA, Singh G. Obesity, abdominal obesity, physical activity, and caloric intake in U.S. Adults: 1988–2010. Am J Med. 2014; 127:717–727. 10.1016/j.amjmed.2014.02.026 24631411 PMC4524881

[2] Huffman MD, Capewell S, Ning H, Shay CM, Ford ES, Lloyd-Jones DM. Cardiovascular health behavior and health factor changes (1988–2008) and projections to 2020: results from the National Health and Nutrition Examination Surveys. Circulation. 2012; 125:2595–2602. 10.1161/CIRCULATIONAHA.111.070722 22547667 PMC3914399

[3] Morrato EH, Hill JO, Wyatt HR, Ghushchyan V, Sullivan PW. Physical activity in U.S. adults with diabetes and at risk for developing diabetes. Diabetes Care. 2007; 30:203–209. 10.2337/dc06-1128 17259482

[4] Wei M, Gibbons LW, Kampert JB, Nichaman MZ, Blair SN. Low cardiorespiratory fitness and physical inactivity as predictors of mortality in men with Type 2 diabetes. Ann Intern Med. 2000; 132:605–611. 10766678 10.7326/0003-4819-132-8-200004180-00002

[5] Myers J, Prakash M, Froelicher V, Do D, Partington S, Atwood JE. Exercise capacity and mortality among men referred for exercise testing. N Engl J Med. 2002; 346:793–801. 10.1056/NEJMoa011858 11893790

[6] Church TS, Cheng YJ, Earnest CP, Barlow CE, Gibbons LW, Priest EL, et al Exercise capacity and body composition as predictors of mortality among men with diabetes. Diabetes Care. 2004; 27:83–88. 14693971 10.2337/diacare.27.1.83

[7] Cooper AR, Sebire S, Montgomery AA, Peters TJ, Sharp DJ, Jackson N, et al Sedentary time, breaks in sedentary time and metabolic variables in people with newly diagnosed type 2 diabetes. Diabetologia. 2012; 55:589–599. 10.1007/s00125-011-2408-x 22167127

[8] Cooper AJ, Brage S, Ekelund U, Wareham NJ, Griffin SJ, Simmons RK. Association between objectively assessed sedentary time and physical activity with metabolic risk factors among people with recently diagnosed type 2 diabetes. Diabetologia. 2014; 57:73–82. 10.1007/s00125-013-3069-8 24196189 PMC3857880

[9] Katzmarzyk PT, Church TS, Craig CL, Bouchard C. Sitting time and mortality from all causes, cardiovascular disease, and cancer. Med Sci Sports Exerc. 2009; 41:998–1005. 10.1249/MSS.0b013e3181930355 19346988

[10] Dunstan DW, Barr EL, Healy GN, Salmon J, Shaw JE, Balkau B, et al Television viewing time and mortality: the Australian Diabetes, Obesity and Lifestyle Study (AusDiab). Circulation. 2010; 121:384–391. 10.1161/CIRCULATIONAHA.109.894824 20065160

[11] Thorp AA, Healy GN, Owen N, Salmon J, Ball K, Shaw JE, et al Deleterious associations of sitting time and television viewing time with cardiometabolic risk biomarkers: Australian Diabetes, Obesity and Lifestyle (AusDiab) study 2004–2005. Diabetes Care. 2010; 33: 327–334. 10.2337/dc09-0493 19918003 PMC2809275

[12] Healy GN, Matthews CE, Dunstan DW, Winkler EA, Owen N. Sedentary time and cardio-metabolic biomarkers in US adults: NHANES 2003–06. Eur Heart J. 2011; 32:590–597. 10.1093/eurheartj/ehq451 21224291 PMC3634159

[13] Colberg SR, Sigal RJ, Fernhall B, Regensteiner JG, Blissmer BJ, Rubin RR, et al Exercise and type 2 diabetes: the American College of Sports Medicine and the American Diabetes Association: joint position statement. Diabetes Care. 2010; 33:e147–167. 10.2337/dc10-9990 21115758 PMC2992225

[14] Katzmarzyk PT. Physical activity, sedentary behavior, and health: paradigm paralysis or paradigm shift? Diabetes. 2010; 59:2717–2725. 10.2337/db10-0822 20980470 PMC2963526

[15] Owen N, Healy GN, Matthews CE, Dunstan DW. Too much sitting: the population health science of sedentary behavior. Exerc Sport Sci Rev. 2010; 38:105–113. 20577058 10.1097/JES.0b013e3181e373a2 PMC3404815

[16] Healy GN, Winkler EA, Owen N, Anuradha S, Dunstan DW. Replacing sitting time with standing or stepping: associations with cardio-metabolic risk biomarkers. Eur Heart J. 2015; 36:2643–2649. 10.1093/eurheartj/ehv308 26228867

[17] Sigal RJ, Kenny GP, Wasserman DP, Castaneda-Sceppa C. Physical activity/exercise and type 2 diabetes. Diabetes Care. 2004; 27:2518–2539. 15451933 10.2337/diacare.27.10.2518

[18] Balducci S, Sacchetti M, Haxhi J, Orlando G, D'Errico V, Fallucca S, et al Physical exercise as therapy for type 2 diabetes mellitus. Diabetes Metab Res Rev. 2014; 30 (Suppl 1):13–23. 24353273 10.1002/dmrr.2514

[19] Korkiakangas EE, Alahuhta MA, Laitinen JH. Barriers to regular exercise among adults at high risk or diagnosed with type 2 diabetes: a systematic review. Health Promot Int. 2009; 24:416–427. 10.1093/heapro/dap031 19793763

[20] Balducci S, Zanuso S, Nicolucci A, De Feo P, Cavallo S, Cardelli P, et al Effect of an intensive exercise intervention strategy on modifiable cardiovascular risk factors in subjects with type 2 diabetes mellitus: a randomized controlled trial: the Italian Diabetes and Exercise Study (IDES). Arch Intern Med. 2010; 170:1794–1803. 21059972 10.1001/archinternmed.2010.380

[21] Di Loreto C, Fanelli C, Lucidi P, Murdolo G, De Cicco A, Parlanti N, et al Validation of a counseling strategy to promote the adoption and the maintenance of physical activity by type 2 diabetic subjects. Diabetes Care. 2003; 26:404–408. 12547870 10.2337/diacare.26.2.404

[22] Nicolucci A, Balducci S, Cardelli P, Cavallo S, Fallucca S, Bazuro A, et al Relationship of exercise volume to improvements of quality of life with supervised exercise training in patients with type 2 diabetes in a randomised controlled trial: the Italian Diabetes and Exercise Study (IDES). Diabetologia. 2012; 55:579–588. 10.1007/s00125-011-2425-9 22234648

[23] Yates T, Davies M, Gorely T, Bull F, Khunti K. Effectiveness of a pragmatic education program designed to promote walking activity in individuals with impaired glucose tolerance: a randomized controlled trial. Diabetes Care. 2009; 32:1404–1410. 10.2337/dc09-0130 19602539 PMC2713638

[24] Kokkinos P, Myers J, Nylen E, Panagiotakos DB, Manolis A, Pittaras A, et al Exercise capacity and all-cause mortality in African American and Caucasian men with type 2 diabetes. Diabetes Care. 2009; 32:623–628. 10.2337/dc08-1876 19196898 PMC2660444

[25] Ekelund U, Ward HA, Norat T, Luan J, May AM, Weiderpass E, et al Physical activity and all-cause mortality across levels of overall and abdominal adiposity in European men and women: the European Prospective Investigation into Cancer and Nutrition Study (EPIC). Am J Clin Nutr. 2015; 101: 613–621. 10.3945/ajcn.114.100065 25733647 PMC4340064

[26] Shephard RJ. Limits to the measurement of habitual physical activity by questionnaires. Br J Sports Med. 2003; 37:197–206. 10.1136/bjsm.37.3.197 12782543 PMC1724653

[27] Healy GN, Dunstan DW, Salmon J, Cerin E, Shaw JE, Zimmet PZ, et al Objectively measured light-intensity physical activity is independently associated with 2-h plasma glucose. Diabetes Care. 2007; 30:1384–1389. 10.2337/dc07-0114 17473059

[28] Balducci S, Sacchetti M, Haxhi J, Orlando G, Zanuso S, Cardelli P, et al The Italian Diabetes and Exercise Study 2 (IDES-2): a long-term behavioral intervention for adoption and maintenance of a physically active lifestyle. Trials. 2015; 16:569 10.1186/s13063-015-1088-0 26651484 PMC4676117

[29] Inoue M, Iso H, Yamamoto S, Kurahashi N, Iwasaki M, Sasazuki S, et al Daily total physical activity level and premature death in men and women: results from a large-scale population-based cohort study in Japan (JPHC study). Ann Epidemiol. 2008; 18:522–530. 10.1016/j.annepidem.2008.03.008 18504139

[30] Sedentary Behaviour Research Network. Letter to the Editor: Standardized use of the terms “sedentary” and “sedentary behaviours”. Appl Physiol Nutr Metab. 2012; 37:540–542. 10.1139/h2012-024 22540258

[31] Balducci S, Zanuso S, Massarini M, Corigliano G, Nicolucci A, Missori S, et al The Italian Diabetes and Exercise Study (IDES): Design and methods for a prospective Italian multicentre trial of intensive lifestyle intervention in people with type 2 diabetic and the metabolic Syndrome. Nutr Metab Cardiovasc Dis. 2008; 18:585–595. 10.1016/j.numecd.2007.07.006 18061415

[32] Herrmann SD, Hart TL, Lee CD, Ainsworth BE. Evaluation of the MyWellness Key accelerometer. Br J Sports Med. 2011; 45:109–113. 10.1136/bjsm.2009.062182 19736173

[33] Bergamin M, Ermolao A, Sieverdes JC, Zaccaria M, Zanuso S. Validation of the MyWellness Key in walking and running speeds. J Sports Sci Med. 2012; 11:57–63. 24149122 PMC3737851

[34] Sieverdes JC, Wickel EE, Hand GA, Bergamin M, Moran RR, Blair SN. Reliability and validity of the Mywellness Key physical activity monitor. Clin Epidemiol. 2013; 5:13–20. 10.2147/CLEP.S38370 23378783 PMC3558248

[35] McGinley SK, Armstrong MJ, Khandwala F, Zanuso S, Sigal RJ. Assessment of the MyWellness Key accelerometer in people with type 2 diabetes. Appl Physiol Nutr Metab. 2015; 40:1193–1198. 10.1139/apnm-2015-0169 26489052

[36] Sacchetti M, Balducci S, Bazzucchi I, Carlucci F, Scotto di Palumbo A, Haxhi J, et al Neuromuscular dysfunction in diabetes: role of nerve impairment and training status. Med Sci Sports Exerc. 2013; 45:52–59. 22843109 10.1249/MSS.0b013e318269f9bb

[37] Penno G, Solini A, Bonora E, Fondelli C, Orsi E, Zerbini G, et al Gender differences in cardiovascular disease risk factors, treatments and complications in patients with type 2 diabetes: the RIACE Italian multicentre study. J Intern Med. 2013; 274:176–191. 10.1111/joim.12073 23565931

[38] Febbraio MA. Exercise and inflammation. J Appl Physiol (1985). 2007; 103:376–377. 17446409 10.1152/japplphysiol.00414.2007

[39] Balducci S, Zanuso S, Nicolucci A, Fernando F, Cavallo S, Cardelli P, et al Anti-inflammatory effect of exercise training in subjects with type 2 diabetes and the metabolic syndrome is dependent on exercise modalities and independent of weight loss. Nutr Metab Cardiovasc Dis. 2010; 20:608–617. 10.1016/j.numecd.2009.04.015 19695853

