# Supplementary material for: Level and correlates of physical activity and sedentary behavior in patients with type 2 diabetes: A cross-sectional analysis of the Italian Diabetes and Exercise Study_2
Source: PLoS One. 2017 Mar 14;12(3):e0173337. doi: 10.1371/journal.pone.0173337 (PMC5349668; doi:10.1371/journal.pone.0173337)
Supplement: S1 File — Diabetes Clinics; Metabolic Fitness Centers; Central laboratory; Data management team; Steering Committee. (DOCX) [file pone.0173337.s001.docx]

**List of participants**

**Diabetes Clinics**

1. Diabetes Unit, Sant’Andrea Hospital, Rome, Italy: Giuseppe Pugliese, Maria Cristina Ribaudo, Martina Vitale, Elena Alessi, Lucilla Bollanti, Francesco G. Conti (Coordinating Center).
2. Diabetes Unit, Fatebenefratelli San Pietro Hospital, Rome, Italy: Nicolina Di Biase, Filomena La Saracina.
3. Diabetes Unit, Health District, Monterotondo, Rome, Italy: Stefano Balducci, Mario Ranuzzi, Jonida Haxhi, Valeria D’Errico.

**Metabolic Fitness Centers**

1. Department of Human Movement and Sport Sciences, ‘‘Foro Italico’’ University, Rome, Italy: Massimo Sacchetti, Giorgio Orlando.
2. Metabolic Fitness Association, Monterotondo, Rome, Italy: Gianluca Balducci, Enza Spinelli.
3. Center for the Study of Metabolism, Rome, Italy: Luca Milo, Roberto Milo.

**Central laboratory**

Laboratory of Clinical Chemistry, Sant’Andrea Hospital, Rome, Italy: Patrizia Cardelli, Stefano Cavallo.

**Data management team**

1. Diabetes Unit, Sant’Andrea Hospital, Rome, Italy: Martina Vitale, Elena Alessi (data control for completeness and plausibility).
2. Department of Human Movement and Sport Sciences, ‘‘Foro Italico’’ University, Rome, Italy: Massimo Sacchetti, Giorgio Orlando; and School of Science, Coventry University, Coventry, UK: Silvano Zanuso (calculation of PA and SED-time).
3. Center for Outcomes Research and Clinical Epidemiology (CORE), Pescara, Italy: Antonio Nicolucci, Giuseppe Lucisano (centralized randomization, data analysis).

**Steering Committee**

Giuseppe Pugliese, Stefano Balducci, Massimo Sacchetti, Silvano Zanuso, Patrizia Cardelli, Antonio Nicolucci.

**Supplemental Tables**

**Supplemental Table 1.** Medication use in the whole cohort and by gender.

| **Variable** | **Total** | **Males** | **Females** | ***P*** |
| --- | --- | --- | --- | --- |
| **n (%)** | 300 (100) | 184 (61.3) | 116 (38.7) |  |
| **Anti-hyperglycemic agents** | 276 (92.0) | 166 (90.2) | 110 (94.8) | 0.152 |
| **Insulin** | 54 (18.0) | 31 (16.8) | 23 (19.8) | 0.513 |
| **Non-insulin agents** | 253 (84.3) | 149 (81.0) | 104 (89.7) | 0.044 |
| **Metformin** | 224 (74.7) | 133 (72.3) | 91 (78.4) | 0.232 |
| **Glitazones** | 39 (13.0) | 23 (12.5) | 16 (13.8) | 0.746 |
| **Sulfonylureas** | 105 (35.0) | 62 (33.7) | 43 (37.1) | 0.551 |
| **Glinides** | 32 (10.7) | 20 (10.9) | 12 (10.3) | 0.886 |
| **DPP-4 inhibitors** | 84 (28.0) | 45 (24.5) | 39 (33.6) | 0.085 |
| **GLP-1 receptor agonists** | 32 (10.7) | 19 (10.3) | 13 (11.2) | 0.810 |
| **Lipid-lowering agents** | 171 (57.0) | 109 (59.2) | 62 (53.4) | 0.324 |
| **Statins** | 141 (47.0) | 86 (46.7) | 55 (47.4) | 0.909 |
| **Ezetimibe** | 16 (5.3) | 11 (6.0) | 5 (4.3) | 0.531 |
| **Fibrates** | 25 (8.3) | 19 (10.3) | 6 (5.2) | 0.116 |
| **Omega-3** | 22 (7.3) | 16 (8.7) | 6 (5.2) | 0.254 |
| **Anti-hypertensive agents** | 222 (74.0) | 129 (70.1) | 93 (80.2) | 0.053 |
| **ACE inhibitors** | 73 (24.3) | 42 (22.8) | 31 (26.7) | 0.444 |
| **Angiotensin receptor blockers** | 125 (41.7) | 71 (38.6) | 54 (46.6) | 0.173 |
| **Calcium-channel blockers** | 62 (20.7) | 40 (21.7) | 22 (19.0) | 0.563 |
| **α-blockers** | 26 (8.7) | 29 (10.3) | 7 (6) | 0.198 |
| **β-blockers** | 74 (24.7) | 36 (19.6) | 38 (32.8) | 0.010 |
| **Diuretics** | 100 (33.3) | 56 (30.4) | 44 (37.9) | 0.180 |
| **Anti-platelet/anti-coagulant agents** | 126 (42.0) | 85 (46.2) | 41 (35.3) | 0.064 |

Values are n (%). GLP-1 = glucagon-like peptide-1; DPP-4 = dipeptidyl peptidase-4; ACE = angiotensin converting enzyme.

**Supplemental Table 2.** Bivariate correlations of LVPA, MVPA, and SED-time with cardiovascular risk factors and scores and physical fitness parameters (Spearman’s rho).

| **Variable** | **LPA** | | **MVPA** | | **SED-time** | |
| --- | --- | --- | --- | --- | --- | --- |
|  | **r** | ***P*** | **r** | ***P*** | **r** | ***P*** |
| **Age** | -0.198 | 0.001 | -0.281 | <0.0001 | 0.152 | 0.008 |
| **Diabetes duration** | -0.153 | 0.008 | -0.178 | 0.002 | 0.167 | 0.004 |
| **HbA_1c_** | -0.305 | <0.0001 | -0.196 | 0.001 | 0.323 | <0.0001 |
| **FPG** | -0.222 | <0.0001 | -0.144 | 0.012 | 0.218 | <0.0001 |
| **Insulin** | -0.109 | 0.059 | -0.100 | 0.084 | 0.139 | 0.016 |
| **HOMA-IR** | -0.180 | 0.002 | -0.137 | 0.018 | 0.207 | <0.0001 |
| **BMI** | -0.248 | <0.0001 | -0.218 | <0.0001 | 0.225 | <0.0001 |
| **Fat mass** | 0.243 | <0.0001 | -0.351 | <0.0001 | 0.227 | <0.0001 |
| **Fat-free mass** | 0.077 | 0.183 | 0.205 | <0.0001 | -0.071 | 0.224 |
| **Waist circumference** | -0.230 | <0.0001 | -0.214 | <0.0001 | 0.200 | <0.0001 |
| **Triglycerides** | -0.111 | 0.055 | -0.127 | 0.027 | 0.135 | 0.019 |
| **Total cholesterol** | 0.031 | 0.596 | 0.074 | 0.199 | -0.026 | 0.657 |
| **HDL cholesterol** | 0.048 | 0.403 | 0.021 | 0.717 | -0.066 | 0.253 |
| **LDL cholesterol** | 0.069 | 0.233 | 0.136 | 0.018 | -0.074 | 0.202 |
| **Systolic BP** | -0.089 | 0.124 | -0.177 | 0.002 | 0.116 | 0.045 |
| **Diastolic BP** | -0.003 | 0.956 | 0.001 | 0.975 | 0.028 | 0.628 |
| **hs-CRP** | -0.226 | <0.0001 | -0.256 | <0.0001 | 0.202 | 0.001 |
| **eGFR** | 0.093 | 0.110 | 0.136 | 0.018 | -0.041 | 0.479 |
| **ACR** | -0.155 | 0.007 | -0.035 | 0.547 | 0.136 | 0.018 |
| **UKPDS CHD risk score** | -0.162 | 0.005 | -0.118 | 0.042 | 0.142 | 0.014 |
| **UKPDS fatal CHD risk score** | -0.206 | <0.0001 | -0.189 | 0.001 | 0.187 | 0.001 |
| **UKPDS stroke risk score** | -0.182 | 0.002 | -0.239 | <0.0001 | 0.148 | 0.010 |
| **UKPDS fatal stroke risk score** | -0187 | 0.001 | -0.256 | <0.0001 | 0.161 | 0.005 |
| **VO_2max_** | 0.586 | <0.0001 | 0.663 | <0.0001 | -0.522 | <0.0001 |
| **Upper body strength** | 0.281 | <0.0001 | 0.397 | <0.0001 | -0.235 | <0.0001 |
| **Lower body strength** | 0.341 | <0.0001 | 0.412 | <0.0001 | -0.299 | <0.0001 |
| **Bending** | -0.143 | 0.013 | -0.107 | 0.064 | 0.142 | 0.014 |
| **LPA** | - | - | 0.591 | <0.0001 | -0.855 | <0.0001 |
| **MVPA** | 0.591 | <0.0001 | - | - | -0.547 | <0.0001 |
| **SED-time** | -0.855 | <0.0001 | -0.547 | <0.0001 | - | - |

LPA = light intensity physical activity; MVPA = moderate-to-vigorous intensity; SED-time = sedentary time; FPG = fasting plasma glucose; HOMA-IR = Homeostasis Model Assessment-Insulin Resistance; BP = blood pressure; hs-CRP = high sensitivity-C-reactive protein; eGFR = estimated glomerular filtration rate; ACR = albumin:creatinine ratio; UKPDS = United Kingdom Prospective Diabetes Study; CHD = coronary heart disease; VO_2max_ = maximal oxygen uptake.
