# Supplementary material for: Level and correlates of physical activity and sedentary behavior in patients with type 2 diabetes: A cross-sectional analysis of the Italian Diabetes and Exercise Study_2
Source: PLoS One. 2017 Mar 14;12(3):e0173337. doi: 10.1371/journal.pone.0173337 (PMC5349668; doi:10.1371/journal.pone.0173337)
Supplement: S1 Table — (DOCX) [file pone.0173337.s002.docx]

**S1 Table.** Medication use in the whole cohort and by gender.

| **Variable** | **Total** | **Males** | **Females** | ***P*** |
| --- | --- | --- | --- | --- |
| **n (%)** | 300 (100) | 184 (61.3) | 116 (38.7) |  |
| **Anti-hyperglycemic agents** | 276 (92.0) | 166 (90.2) | 110 (94.8) | 0.152 |
| **Insulin** | 54 (18.0) | 31 (16.8) | 23 (19.8) | 0.513 |
| **Non-insulin agents** | 253 (84.3) | 149 (81.0) | 104 (89.7) | 0.044 |
| **Metformin** | 224 (74.7) | 133 (72.3) | 91 (78.4) | 0.232 |
| **Glitazones** | 39 (13.0) | 23 (12.5) | 16 (13.8) | 0.746 |
| **Sulfonylureas** | 105 (35.0) | 62 (33.7) | 43 (37.1) | 0.551 |
| **Glinides** | 32 (10.7) | 20 (10.9) | 12 (10.3) | 0.886 |
| **DPP-4 inhibitors** | 84 (28.0) | 45 (24.5) | 39 (33.6) | 0.085 |
| **GLP-1 receptor agonists** | 32 (10.7) | 19 (10.3) | 13 (11.2) | 0.810 |
| **Lipid-lowering agents** | 171 (57.0) | 109 (59.2) | 62 (53.4) | 0.324 |
| **Statins** | 141 (47.0) | 86 (46.7) | 55 (47.4) | 0.909 |
| **Ezetimibe** | 16 (5.3) | 11 (6.0) | 5 (4.3) | 0.531 |
| **Fibrates** | 25 (8.3) | 19 (10.3) | 6 (5.2) | 0.116 |
| **Omega-3** | 22 (7.3) | 16 (8.7) | 6 (5.2) | 0.254 |
| **Anti-hypertensive agents** | 222 (74.0) | 129 (70.1) | 93 (80.2) | 0.053 |
| **ACE inhibitors** | 73 (24.3) | 42 (22.8) | 31 (26.7) | 0.444 |
| **Angiotensin receptor blockers** | 125 (41.7) | 71 (38.6) | 54 (46.6) | 0.173 |
| **Calcium-channel blockers** | 62 (20.7) | 40 (21.7) | 22 (19.0) | 0.563 |
| **α-blockers** | 26 (8.7) | 29 (10.3) | 7 (6) | 0.198 |
| **β-blockers** | 74 (24.7) | 36 (19.6) | 38 (32.8) | 0.010 |
| **Diuretics** | 100 (33.3) | 56 (30.4) | 44 (37.9) | 0.180 |
| **Anti-platelet/anti-coagulant agents** | 126 (42.0) | 85 (46.2) | 41 (35.3) | 0.064 |

Values are n (%). GLP-1 = glucagon-like peptide-1; DPP-4 = dipeptidyl peptidase-4; ACE = angiotensin converting enzyme.
